# Psychosocial Interventions in Reducing Anxiety and Depression in CABG Patients: A Systematic Review and Meta-Analysis

**DOI:** 10.1192/j.eurpsy.2026.10625

**Published:** 2026-07-31

**Authors:** E. H. Kozan Çıkırıkçı, N. D. Kılınç, M. N. Tümkaya, Z. I. Kılınç, S. Tümkaya

**Affiliations:** 1 School of Nursing; 2 Institute of Health Sciences, Koç University; 3 Cardiovascular Surgery, Dr Sadi Konuk Education and Research Hospital, İstanbul; 4 Cardiovascular Surgery, Şanlıurfa Education and Research Hospital, Şanlıurfa, Türkiye

## Abstract

**Introduction:**

Coronary artery bypass grafting (CABG) is among the most frequently performed interventions globally for the treatment of coronary artery disease (CAD). Anxiety and depression are common among patients undergoing coronary artery bypass graft (CABG) surgery and are associated with poorer recovery and quality of life.

**Objectives:**

Psychosocial interventions are increasingly used to address mental health needs in cardiac populations, but evidence regarding their effectiveness in CABG patients remains limited and inconsistent. This study sought to clarify their impact through systematic review and meta-analysis.

**Methods:**

A systematic review and meta-analysis were conducted in accordance with PRISMA guidelines and registered in PROSPERO database (No: CRD420251022254). Randomized controlled trials (RCTs) published within the last 10 years were retrieved from nine databases. Eligible studies evaluated psychosocial interventions targeting anxiety and/or depression in CABG patients. Risk of bias was assessed using the Cochrane RoB 2 tool. A random-effects model was used to calculate pooled effect sizes (Hedges’ g).

**Results:**

Seven RCTs (n = 1,371) were included. Interventions comprised psychoeducation, web-based support, and relaxation-based methods. Meta-analysis showed a significant reduction in anxiety symptoms (Hedges’ g = –0.51, *p* = .004; I² = 72.9%), indicating a moderate effect. For depression, the pooled effect was not statistically significant (Hedges’ g = –0.38, *p* = .059; I² = 51.4%), although some studies reported delayed or subgroup-specific improvements.

**Conclusions:**

Psychosocial interventions appear effective in reducing anxiety in CABG patients, while their impact on depression is less clear and may depend on longer-term or tailored approaches. The evidence supports a paradigm shift toward integrated, multidisciplinary care for CABG patients, where psychosocial health is addressed as a core component of recovery alongside physical rehabilitation.Further rigorous trials are needed to establish their role in routine post-CABG care.

**Disclosure of Interest:**

None Declared

